# Structural and functional insights from the sequences and complex domain architecture of adhesin-like proteins from *Methanobrevibacter smithii* and *Methanosphaera stadtmanae*

**DOI:** 10.3389/fmicb.2024.1463715

**Published:** 2024-10-21

**Authors:** Anjali Bansal Gupta, Henning Seedorf

**Affiliations:** ^1^Temasek Life Sciences Laboratory Limited, 1 Research Link National University of Singapore, Singapore, Singapore; ^2^Department of Biological Sciences, National University of Singapore, Singapore, Singapore

**Keywords:** adhesins, methanogens, adhesin-like proteins (ALPs), archaeal big domain, gut micobiome

## Abstract

Methanogenic archaea, or methanogens, are crucial in guts and rumens, consuming hydrogen, carbon dioxide, and other fermentation products. While their molecular interactions with other microorganisms are not fully understood, genomic sequences provide information. The first genome sequences of human gut methanogens, *Methanosphaera stadtmanae* and *Methanobrevibacter smithii*, revealed genes encoding adhesin-like proteins (ALPs). These proteins were also found in other gut and rumen methanogens, but their characteristics and functions remain largely unknown. This study analyzes the ALP repertoire of *M. stadtmanae* and *M. smithii* using AI-guided protein structure predictions of unique ALP domains. Both genomes encode more than 40 ALPs each, comprising over 10% of their genomes. ALPs contain repetitive sequences, many of which are unmatched in protein domain databases. We present unique sequence signatures of conserved ABD repeats in ALPs and propose a classification based on domain architecture. Our study offers insights into ALP features and how methanogens may interact with other microorganisms.

## Introduction

Methanogens play an important role in intestinal tracts and rumens as consumers of hydrogen, carbon dioxide, and other end products of bacterial and eukaryotic fermentations. The biochemistry and bioenergetics of methanogenic archaea have been the subjects of research for several decades (Thauer et al., 2008), but the molecular interactions of methanogens with their environment, specifically with other organisms, remain largely unknown. The genome sequences of some methanogens do provide potential cues in this regard as they indicate the presence of adhesin-like proteins (ALPs) in these microorganisms.

Adhesin-like proteins were discovered and annotated as “asn/thr-rich large proteins” in the genome of *Methanosphaera stadtmanae*, the first genome-sequenced human gut methanogen (Fricke et al., 2006). At the time of discovery, no close homologs of ALPs were found in other archaeal genomes or public databases. However, these proteins had several characteristic features: (a) an overrepresentation of asparagine and threonine in the protein sequences; (b) the length of ALPs often vastly exceeded the mean protein length in *M. stadtmanae*; (c) repetitive primary sequence motifs of variable length; and (d) most of the ALPs of *M. stadtmanae* were predicted to be anchored N-terminally in the membrane and pointed to the extracellular space. Due to the habitat, it was assumed that the function of these proteins may be relevant to the commensal lifestyle of *M. stadtmanae* (Fricke et al., 2006). This assumption was further corroborated when the subsequently sequenced genome of the human gut methanogen, *Methanobrevibacter smithii*, revealed the presence of 48 protein homologs of asn/thr-rich large proteins (Samuel et al., 2007). Many of the *M. smithii* ALPs showed similarities to the ALPs of *M. stadtmanae*, but some ALPs appeared to be specific *to M. smithii* or *M. stadtmanae*. In addition, Samuel et al. (2007) identified features in some ALPs that are found in bacterial adhesins and named therefore these proteins “adhesin-like proteins.” The name “ALP” was subsequently used by most others when annotating these types of genes. However, there is no clear definition of what a methanogen ALP constitutes. Nonetheless, ALPs have since been annotated in the genomes of many other methanogens, primarily belonging to the *Methanobacteriales* and *Methanomassillicoccales*, many of which are found in the intestinal tract of animals and humans (Borrel et al., 2017; de la Cuesta-Zuluaga et al., 2021; Leahy et al., 2013; Li et al., 2016; Poehlein et al., 2017; Poehlein and Seedorf, 2016). The exact functions of most ALPs remain speculative, and only a few studies have experimentally investigated ALPs in more detail. In a study by Hansen et al., it was shown that *M. smithii* ALPs may be differentially expressed, depending on the presence of growth substrates, e.g., formate (Hansen et al., 2011). The only experimental evidence that ALPs could potentially act as actual adhesins was obtained in a phage display experiment, which could show that the display of a *Methanobrevibacter ruminantium* ALP domain in rumen extract enabled the enrichment of some protozoa and bacterial microorganisms (Ng et al., 2016). However, the exact mechanism for this enrichment remains to be elucidated, as well as many other features of ALPs. The limited knowledge about the structure and function of ALPs is partially due to the long length of these proteins and the current lack of genetic tools to genetically manipulate the methanogens of interest, specifically for *Methanobacteriales* and *Methanomassilicoccales* species.

In recent years, protein databases have substantially increased, especially with the advent of artificial intelligence-supported programs for protein structure predictions like AlphaFold (Jumper et al., 2021), which have greatly increased the ability to predict tertiary protein structures. In this study, we leverage these recent developments and analyze in detail the structural architectures of 91 ALPs from two major human gut methanogen symbionts, *M. stadtmanae* and *M. smithii*. Furthermore, we identify common features of well-characterized bacterial adhesins with archaeal ALPs, propose a classification of *M. smithii* and *M. stadtmanae* ALPs, and finally, discuss potential mechanisms on how ALPs may interact with their targets.

## Results

### Detection of adhesin-like proteins in *Methanobrevibacter smithii* and *Methanosphaera stadtmanae*

Adhesin-like proteins have complex and diverse sequences with variable lengths. There are currently no comprehensive databases for the annotation of ALPs in methanogen genomes, and ALPs have, in general, not been defined. In this study, we applied an expanded search approach using annotated ALPs from 13 other methanogens (refer to materials and methods) to query the genomes of *M. stadtmanae* and *M. smithii*. We report 49 ALPs in the genome of *M. smithii* and 42 in the *M. stadtmanae* genome, which is more than that reported in earlier studies (Fricke et al., 2006; Samuel et al., 2007; Hansen et al., 2011). Some ALPs were previously annotated as hypothetical proteins and were therefore not reported at the time the genomes were published. The 91 ALPs in the two species reported here account for ~10% of the genome of *M. smithii* and *M. stadtmanae* each (Table 1). This fraction is significantly larger in comparison to bacteria, where only <1.5% of the proteome is dedicated to code for adhesins (Monzon et al., 2021). This difference is mainly due to the higher number of ALP genes in methanogens as compared to those in bacteria (Monzon et al., 2021). The lengths of ALPs ranged from 128 amino acids to 4,691 amino acids in *M. smithii* (average: 1,299 amino acids) and 251 amino acids to 3,356 amino acids in *M. stadtmanae* (average: 1,462 amino acids). Both the number and the length of ALPs contribute toward the large fraction of genome coding for ALPs in two species.

**Table 1:** Summary of ALPs in *Methanobrevibacter smithii* and *Methanosphaera stadtmanae.*

S.No.	Name	Genome size (Mbp)	Proteome accession	Number of ALPs	ALP coding proteome (aa)	Fraction of ALP coding genome (%)
1.	*M. smithii*	1.853	NC_009515	49	67,292	10.9
2.	*M. stadtmanae*	1.767	NC_007681	42	63,039	10.7

### Identification and characterization of protein domains in *Methanobrevibacter smithii* and *Methanosphaera stadtmanae* ALPs

**Figure 1:**
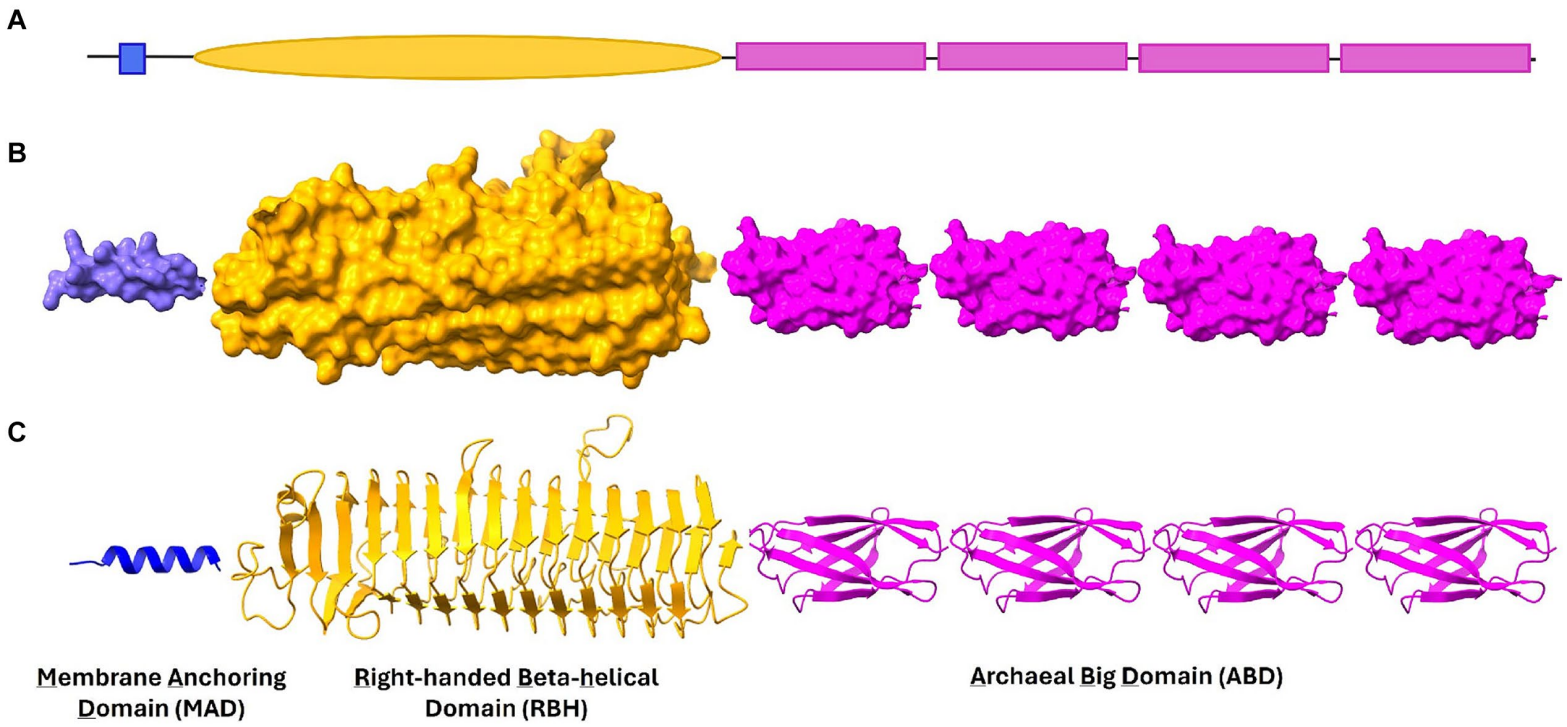
Domains in *Methanobrevibacter smithii* and *Methanosphaera stadtmanae* ALPs. A typical ALP consists of Membrane Anchoring Domain (MAD), Right-handed beta helical domain (RBH) and Archaeal Big domain (ABD) (shown in schematic representation in **A**). These three domains are shown in **(B)** surface and **(C)** ribbon representation in this figure. In general, ABD domains are found in repeats in most ALPs and together with RBH they present binding sites for unique molecules on surface of other microbes.

Functional annotations using reference-based tools available in InterPro and Pfam (Mistry et al., 2020; Blum et al., 2021; Paysan-Lafosse et al., 2022) indicate an absence of domains in a large part of ALP sequences or only limited similarity of domains with Pfam references. Only 30 *M. smithii* and 24 *M. stadtmanae* ALPs could be matched to at least one domain in Pfam, which is now part of the InterPro database. We assigned domains to ALPs based on sequence similarity to the nearest proteins in the AlphaFold database (Supplementary Table 1). It was found that archaeal ALPs from the two methanogens have mainly three to four different domain types: membrane-anchoring domain (s) (MAD), archaeal big domain (ABD), and right-handed beta-helical (RBH) domain, while other domains such as transglutaminase-like domain (TG-like) and carbohydrate-binding domains were also detectable in some ALPs (Figure 1). The frequency of occurrence of the different domains in ALPs is shown in Table 2. The detailed features of these domains are presented in the sections below.

**Table 2:** Domains in ALPs of *Methanobrevibacter smithii* and *Methanosphaera stadtmanae*.

S.No.	*Domain annotations (Pfam)*	Number of ALPs with these domains*	
		*M. smithii*	*M. stadtmanae*
1	Membrane-anchoring domain	47	39
2	Archaeal big domain (ABD)	45	40
3	Right-handed beta-helical domain (RBH)	28	34
4	Transglutaminase-like superfamily	2	3
5	Pseudomurein-binding repeat	2	3
6	Carboxypeptidase regulatory-like domain	1	3
7	Chlamydia polymorphic membrane protein (Chlamydia_PMP) repeat	1	2
8	Papain family cysteine protease	1	1
9	PQQ-like domain repeats	-	1
10	Peptidase propeptide and YPEB domain repeats	-	1
11	Putative glycosyl hydrolase domain	1	-

^*^Functional domain annotations are taken from Pfam database except for RBH and ABD domains. AlphaFold structures were referred to confirm for presence of ABD and RBH domains only.

### Membrane-anchoring domain in *Methanobrevibacter smithii* and *Methanosphaera stadtmanae* ALPs

Two types of MADs were recognized in ALPs of two organisms, transmembrane (TM) *α*-helices and amphipathic helices. TM helices were present in most ALPs of both organisms. In general, TM helices are present at the N-terminus of ALPs; in some cases, e.g., YP_001272624 of *M. smithii,* a duplication of the TMH at the N-terminus, can be observed. In 11 *M. smithii* ALPs, MADs were present at both termini, which may indicate further complexity of potential interaction with other microbes. In comparison, *M. stadtmanae* had a single N-terminal MAD in 39 out of 42 ALPs, and three ALPs had no MAD.

For some ALP sequences (10 in *M. smithii*, four in *M. stadtmanae*), we noticed the presence of N-or C-terminal helices with hydrophobic residues in the AlphaFold structure; however, TMHMM failed to assign them any transmembrane (TM) domain. These were < 20-amino-acid-long sequences of hydrophobic residues, which might not form a complete TM helix (Supplementary Table 2). The typical length of the TM helix was suggested to be 24.0 (±5.6) amino acids (Baeza-Delgado et al., 2013) though it depends on the amino acid sequence and hydrophobicity (Krishnakumar and London, 2007). We noticed that the hydrophobicity index values of short hydrophobic helices in ALPs typically were > 1, which was comparable to the values determined for full-length TM helices using the HeliQuest sequence analysis module (Gautier et al., 2008). These short helices were rich in long-chain hydrophobic residues such as leucine, isoleucine, and phenylalanine (Kyte and Doolittle, 1982). Together with TM helical domains, we marked these short helices as membrane-anchoring domains as they might also help anchor ALPs to the lipid membrane. Earlier studies have shown that the lipid bilayer can adapt to TM helices as short as 10–12 leucines (Baeza-Delgado et al., 2016) and can adjust to negative mismatches (Krishnakumar and London, 2007). It is proposed that the response of short helices to the surrounding lipid bilayer depends on the nature of lipids the helix is in contact with, its amino acid composition, and the distribution of amino acids along the helix. The lipid bilayer may respond to hydrophobic mismatch caused by short helices by compression and chain disordering (de Planque et al., 1998). Furthermore, the packing response of lipids around the single helix could be different than that for larger proteins (Weiss et al., 2003). Marginally hydrophobic *α*-helices have also been shown useful in membrane protein folding (De Marothy and Elofsson, 2015).

In addition to the short membrane-anchoring α-helices, we also observed the presence of amphipathic helices at the terminals in the structures of some ALPs, where in most cases a TM helix was missing. Using helical wheel diagrams (Supplementary Figure 1), we showed that such sequences in ALPs could fold into an amphipathic α-helix with a hydrophobic face and a hydrophobicity index >50%, as predicted by HeliQuest (Gautier et al., 2008). It is possible to assume that such helices could anchor ALPs to a lipid membrane by lying parallel to a lipid bilayer membrane with the hydrophilic surface interacting with charged lipid head groups while the hydrophobic side is exposed to the fatty acid chains of a membrane lipid. Almost all ALPs had at least one MAD except five ALPs (YP_001272625 and YP_001274282 from *M. smithii* and YP_447499, YP_447699, and YP_447953 from *M. stadtmanae*). This could be due to the partial annotations of these proteins from the genomic sequence, or it is also possible that the MAD could not be predicted with given algorithms.

Furthermore, ALPs are also found to be rich in asparagine/lysine/arginine residues at the N-terminus of the transmembrane domain. The cytoplasmic di-lysine motifs shown to be involved in the trafficking of protein to the ER and plasma membrane in earlier studies on eukaryotic cells (Chau et al., 2021; Jackson et al., 2012) were observed at the N-terminus of transmembrane helices in 22 ALPs, and 36 other ALPs had one of the lysine substituted by asparagine, while others also had arginine and aspartic acid (Supplementary Table 3). In ALPs, such motifs might be important for the protein to insert a MAD into the membrane in the required orientation. Interestingly, such motifs were also observed near the C-terminal TM helix, indicating their possible orientation to be on the cytoplasmic side (Supplementary Table 4). The presence of such signals adjacent to TM helices gives information about the possible orientation of helices in cell membranes such as *M. smithii*’s YP_001272624, which has two TM helices at the N-terminus. The di-lysine motif was found only before the second TM helix, indicating its possible orientation from cytoplasmic to the extracellular side. It is to be noted that such signal sequences were missing in most short helices, suggesting that small helices could help in insertion although not spanning the whole membrane bilayer.

### Right-handed beta-helical domain in *Methanobrevibacter smithii* and *Methanosphaera stadtmanae* ALPs

Right-handed beta-helical is the third most represented domain found in ALPs of the two methanogens (Table 2). *Methanobrevibacter smithii* had 28 ALPs with 43 RBH domains, and *M. stadtmanae* has 34 ALPs with 52 RBH domains. Many ALPs had repeats of the RBH domain such as YP_001273761 of *M. smithii*, which had seven repeats.

The RBH domain was initially identified in *Erwinia chrysanthemi*, a plant pathogenic bacterium as a pectin-binding domain of pectate lyase C (Yoder et al., 1993), and subsequently has been discovered in many other enzymatic proteins such as those involved in hydrolyzing lectins and other carbohydrates (Jenkins and Pickersgill, 2001). The beta-helical rod of this parallel *β*-helical domain provides a larger groove on its surface for recognizing long carbohydrate molecules (Suits and Boraston, 2013; Villarreal et al., 2022). This is mediated through the chain of conserved asparagines in the loops, which has been suggested to be the most common amino acid in RBH domains (Iengar et al., 2006). Loops mainly have charged and polar amino acids, which explains their ability to bind to long polysaccharides. InterPro entry (IPR039448) indicates that the RBH domain is highly represented in bacteria as compared to other groups of organisms, while in Archaea, it is mostly found in *Methanobacteriaceae* and *Methanosarcinaceae* families. Particularly for the former, this could be due to their presence in ALPs, which form a large fraction of the archaeal proteome. Similar structures have also been observed in viruses for the purpose of host attachment and infection (Weigele et al., 2003).

The fold was suggested to have diverged from a common ancestor based on the presence of conserved alpha-helix capping the N-terminus of beta-helix. The cap motif also inhibits oligomeric interactions similar to those found in amyloid formations (Bryan et al., 2011). Furthermore, it is noted that RBH domains in archaeal ALPs are relatively large. The longest RBH domain (YP_447868 of *M. stadtmanae*) with a length of 1,236 amino acid folds into ~100 Å long RBH domain rod carrying 14 turns. Although rare in eukaryotes, the RBH domain is highly prevalent in surface proteins of bacteria and fungi, with many of them involved in pathogenesis (Bradley et al., 2001).

### Archaeal big domain

**Figure 2:**
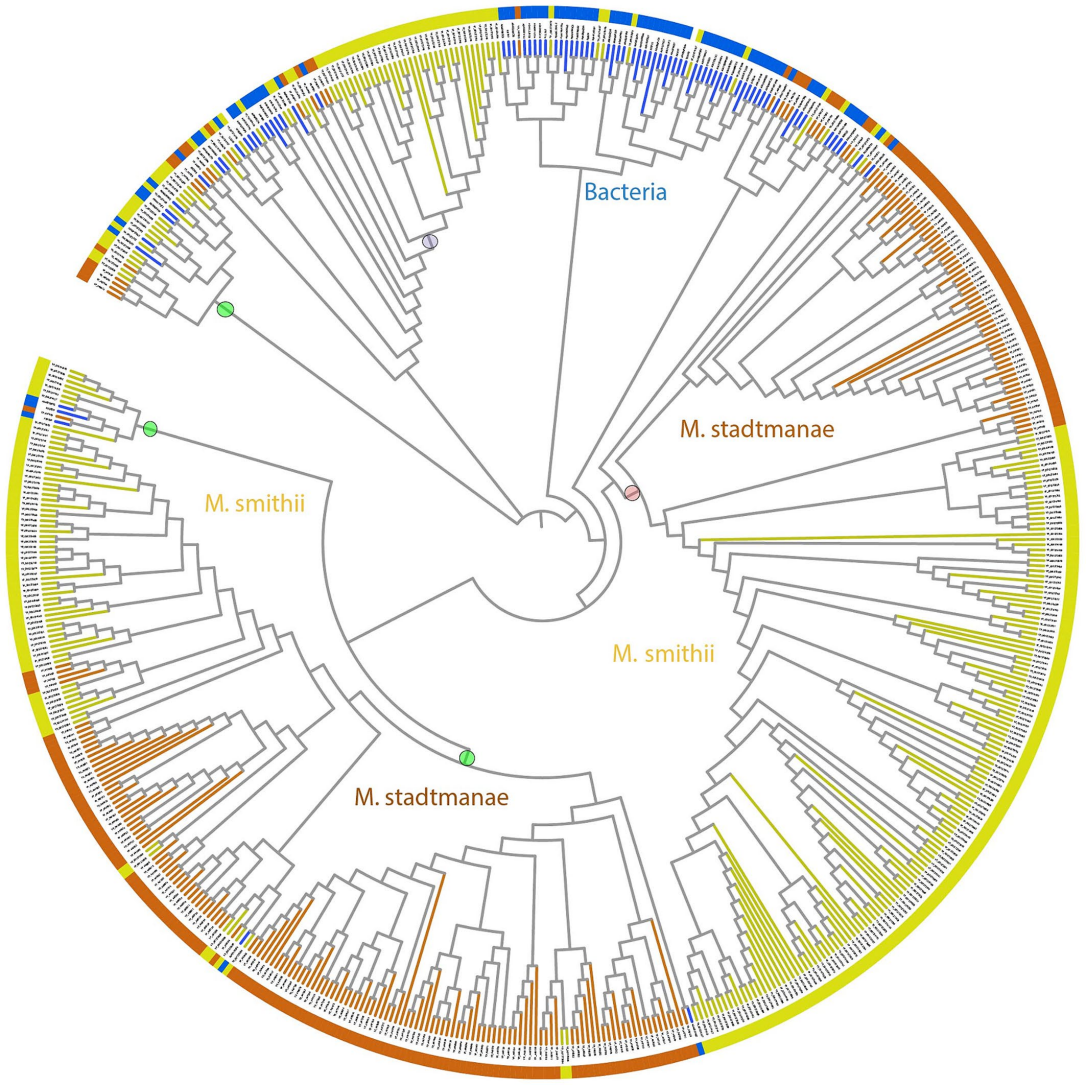
Phylogenetic tree of stalk domains from bacteria together with archaeal big domains (ABDs) of *Methanobrevibacter smithii* and *Methanosphaera stadtmanae*. This bootstrapped neighbor-joining tree is constructed using 279 ABD domains belonging to 49 *M. smithii* ALPs, 222 ABD domains of 42 *M. stadtmanae* ALPs, and 80 stalk domains from bacteria, as described in a previous study (Monzon et al., 2021). The tree is displayed in circular mode with branch lengths ignored to maintain the clarity of display. Branches corresponding to *M. smithii* and *M. stadtmanae* ABDs are marked in yellow and brown, respectively, whereas bacterial stalk domain branches are in blue. The phylogenetic tree shows the distinct clustering of archaeal ABDs from bacterial stalk domains. ABDs group in a number of clusters as marked with circles against the branches. The clade marked with a purple circle has nine β-stranded ABDs, while the clade marked with a pink circle was used to create WebLogo (Figure 3). Each clade has ALPs belonging to *M. smithii* and *M. stadtmanae,* and we believe that more ABD clades will be known as more ALPs are studied in other archaeal species.

Archaeal big domains are the most abundant domain repeats in *M. smithii* and *M. stadtmanae* ALPs and are found in almost all the ALP sequences. Figure 2 depicts the phylogenetic tree constructed based on the alignment of 279 *M. smithii* ABDs and 222 *M. stadtmanae* ABDs with 80 bacterial stalk domains (Monzon et al., 2021). These ABDs seem to have diverged from bacterial stalk domains as most of the archaeal sequences cluster together in a phylogenetic tree and form groups distinct from the bacterial stalk domain sequences. The archaeal big domain (ABD) definitions are not included in the Pfam domain database (Mistry et al., 2020). Pfam either failed to assign domain family to a large part of archaeal ALPs or assigned Big3 (Pfam ID: PF16640) and DUF11 (Pfam ID: PF01345) domains in most cases, generally with high e-value. We also searched the ABD domains in “refseq_genomes (1,711 databases) at NCBI, excluding archaea (NCBI taxid: 2,157)”, and found no blast hit, indicating that these domains may be unique to archaeal species and are not ubiquitously found in other domains of life, suggesting their potential specific role in archaeal symbiosis.

We noticed broad clades of ABD domains in *M. smithii* and *M. stadtmanae,* as marked in Figure 2. Most clades contained no sequences of apparent bacterial origin; some ABD clades clustered with at least four bacterial stalk domain sequences (MucBP (A0A806LF85), LVIVD (A0A0S1YA82), and Trp_ring (F9N556) of non-ESET clan and Big6 (A0A150KJ36), Big3_5 (A0A2V7S5F5), Big3 (R5U8D9), and Big2 (A0A0E1X8Y2) of ESET clan) in the phylogenetic tree (Figure 2). These could be the precursors from which archaeal ABDs evolved and subsequently diverged to acquire unique features. Some ABDs were longer as compared to the most common ABD domains, and only a few ALPs had such domains (YP_001272746, YP_001272984, YP_001274106, and YP_001274107 of *M. smithii*) (Supplementary Figure 2).

**Figure 3:**
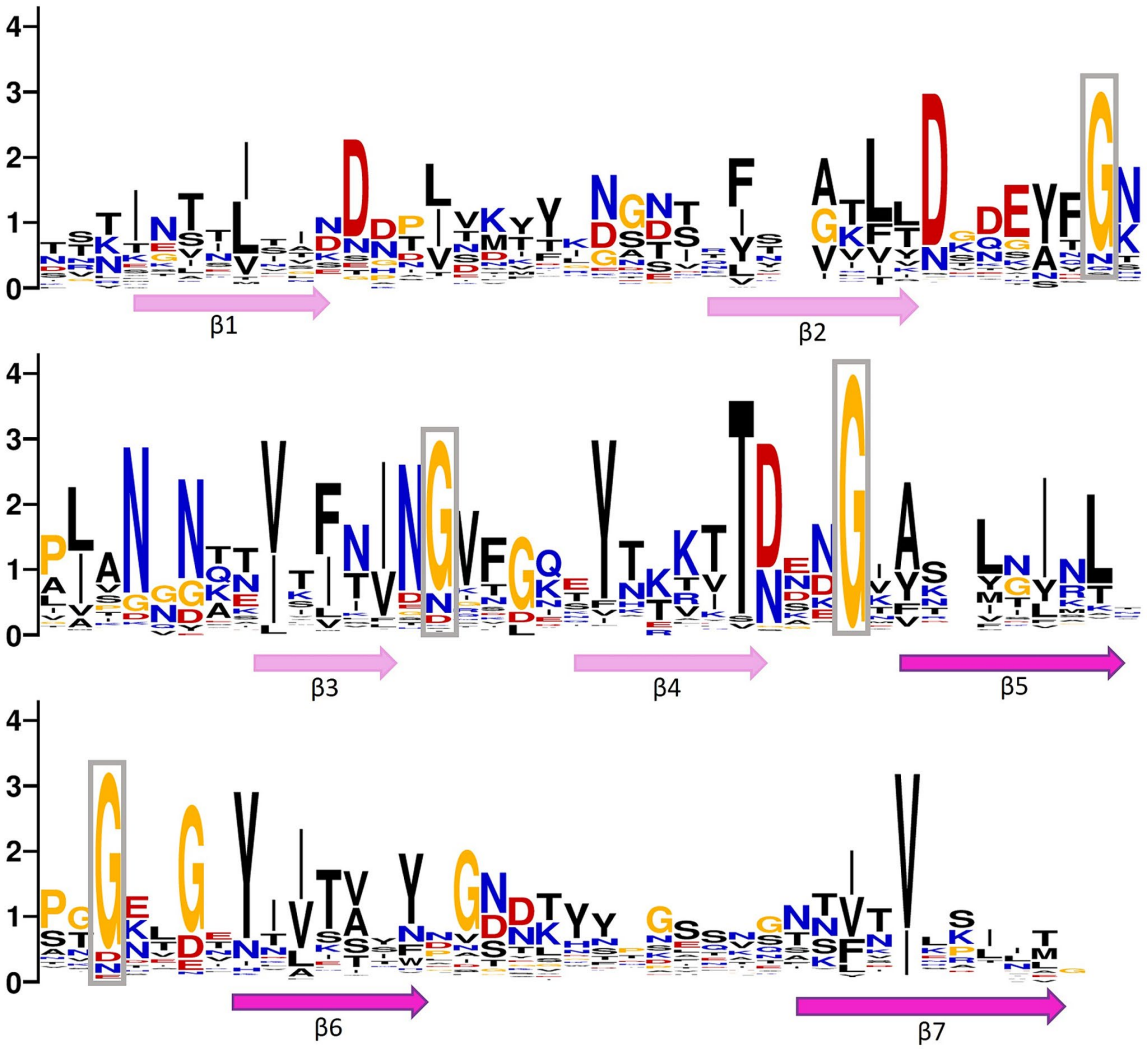
Positional entropy of archaeal big domain (ABD). WebLogo is created using multiple sequence alignment of ABDs belonging to both *Methanobrevibacter smithii* and *Methanosphaera stadtmanae* (clade marked with a pink circle in Figure 2). Hydrophobic residues (phenylalanine, tyrosine, leucine, isoleucine, glycine, valine, and alanine) are marked in black, positively charged amino acids (lysine, arginine, asparagine, and glutamine) are in blue, negatively charged amino acids (aspartic acid and glutamic acid) are marked in red, and threonine and cysteine are in yellow. The relative position of beta strands is also marked below the WebLogo. The lengths of strands and loops are not scaled. The long gaps present in alignment were deleted before creating a logo. The highly conserved glycine residues in loops are boxed.

We analyzed the frequency and patterns of amino acids present in ABDs and bacterial stalk domains (Figure 3). The comparison clearly shows that although archaeal ABDs have glycine residues conserved similar to bacterial stalk domains, they also acquired unique features with high conservation. The uniquely conserved residues of archaeal ABDs are marked in Figure 3.

**Figure 4:**
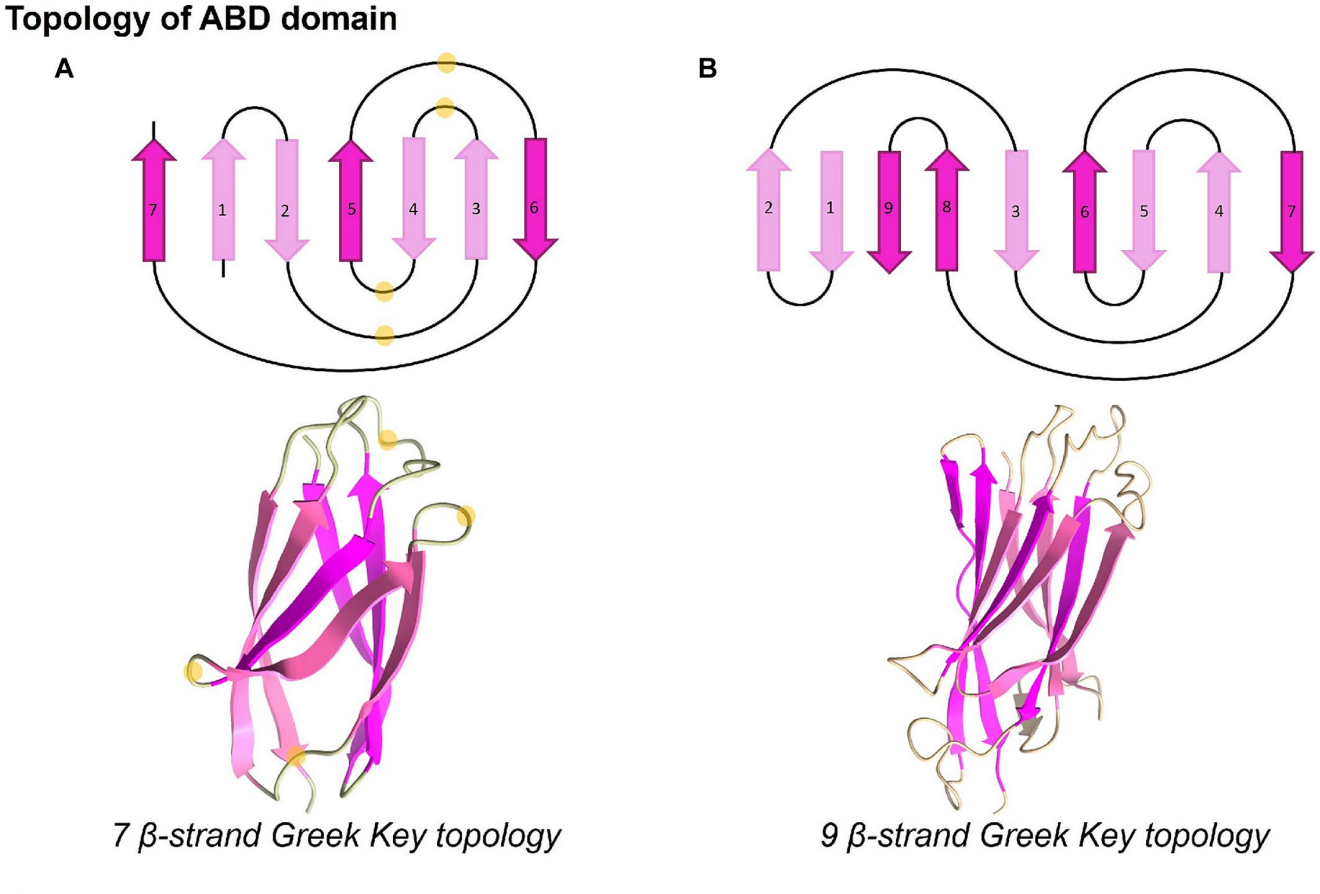
Topology and three-dimensional structures of ABD domains. ABD domains present distinct Greek-key topologies and fold into seven or nine beta-stranded conformations. The highly conserved glycine residues present in loops, as seen in WebLogo, are marked with light yellow circles. The structures were displayed using UCSF ChimeraX version 1.5 (Pettersen et al., 2021).

An ABD folds into a typical *β*-sandwich in Greek-key topology with seven strands (Figure 4A). A β-sandwich domain of longer ABDs is formed by nine β-strands (Figure 4B). The conserved glycine residues present in loops are marked in the representative three-dimensional structure obtained from AlphaFold, as shown in Figure 4A. Notably, the conserved residues occur in loops, which may be important for interaction with other protein domains, while the core is conserved with hydrophobic residues. Furthermore, we noticed that some strands in ABD folds have conserved long-chain hydrophobic residues such as valine, isoleucine, phenylalanine, and leucine, while others show conservation of smaller residues such as glycine. The representative structure was searched in the Dali database to identify the closest structural homolog of ABD. It is interesting to note that the root mean squared deviation (r.m.s.d) of ABD of *M. smithii* (NCBI accession: YP_001272624) with the nearest structure (Big1 domain of bacterial invasion, PDB ID: 1CWV) was only 1.8 Å, while they shared only 10% sequence similarity. Similarly, the nearest structural homolog of another ABD domain of the same ALP belonging to a different clade in the phylogenetic tree (HLA Class-I Histocompatibility antigen, PDB ID: 1EWO, r.m.s.d.: 1.8 Å) was only 18% similar. This indicated sequence divergence from bacterial ancestral homologs while conserving the overall three-dimensional fold of seven β-strands.

Archaeal big domain is found in repeats on ALPs and May be important for extending the range to reach symbiotic microbes. ABD repeats of some ALPs are highly similar; for example, YP_447631 of *M. stadtmanae* has 27 ABDs belonging to two major clades in a phylogenetic tree, and 20 of them share 60.6% average similarity with each other, while some other ALPs have divergent ABD sequences belonging to other phylogenetic clades; for example, YP_447476 of *M. stadtmanae* has three ABDs, and all of them cluster with three different clades, indicating early divergence of ABDs from bacteria.

### Other domains

Less common domain types in the ALPs of *M. smithii* and *M. stadtmanae* are currently mostly grouped under ‘others’ as being different from the commonly observed MAD, RBH, and ABD domain types. While the overall occurrence of these domain types is low in ALPs of these two methanogens, it is observed that these domains vary and, in some ALPs, multiple ‘others’ can be detected. These can comprise domains that show limited structural similarity to domains such as transglutaminase, pseudomurein-binding protein, PQQ-like domain, and lectin-like domain. These are listed in Table 2, along with the number of occurrences in ALPs of two organisms.

### Groups within *Methanobrevibacter smithii* and *Methanosphaera stadtmanae* ALPs

Functional domain annotations using Pfam indicated that *M. smithii* and *M. stadtmanae* ALPs are less diverse as compared to bacteria. Only 11 domain families were identified from a sequence-based search in the Pfam database in both species together. On the other hand, in bacteria, altogether 109 types of stalk and adhesive domains are present (Monzon et al., 2021; Monzon and Bateman, 2022). The most common architecture in analyzed archaeal ALPs was RBH domain repeats at the N-terminus, followed by ABD domain repeats. A single transmembrane helix at the N-terminus in the majority of ALPs could act as a membrane-anchoring domain; however, in others, it was present at the C-terminus or at both ends. In addition to the above ALPs, there were 14 sequences in *M. smithii* and *M. stadtmanae,* respectively, that had missing RBH and ABD domains, although these were picked in our sequence-based search. These could be partial as they are short and could likely present incomplete domains. These sequences were discarded and not classified as ALPs in this study (Supplementary Tables 5A,B).

**Figure 5:**
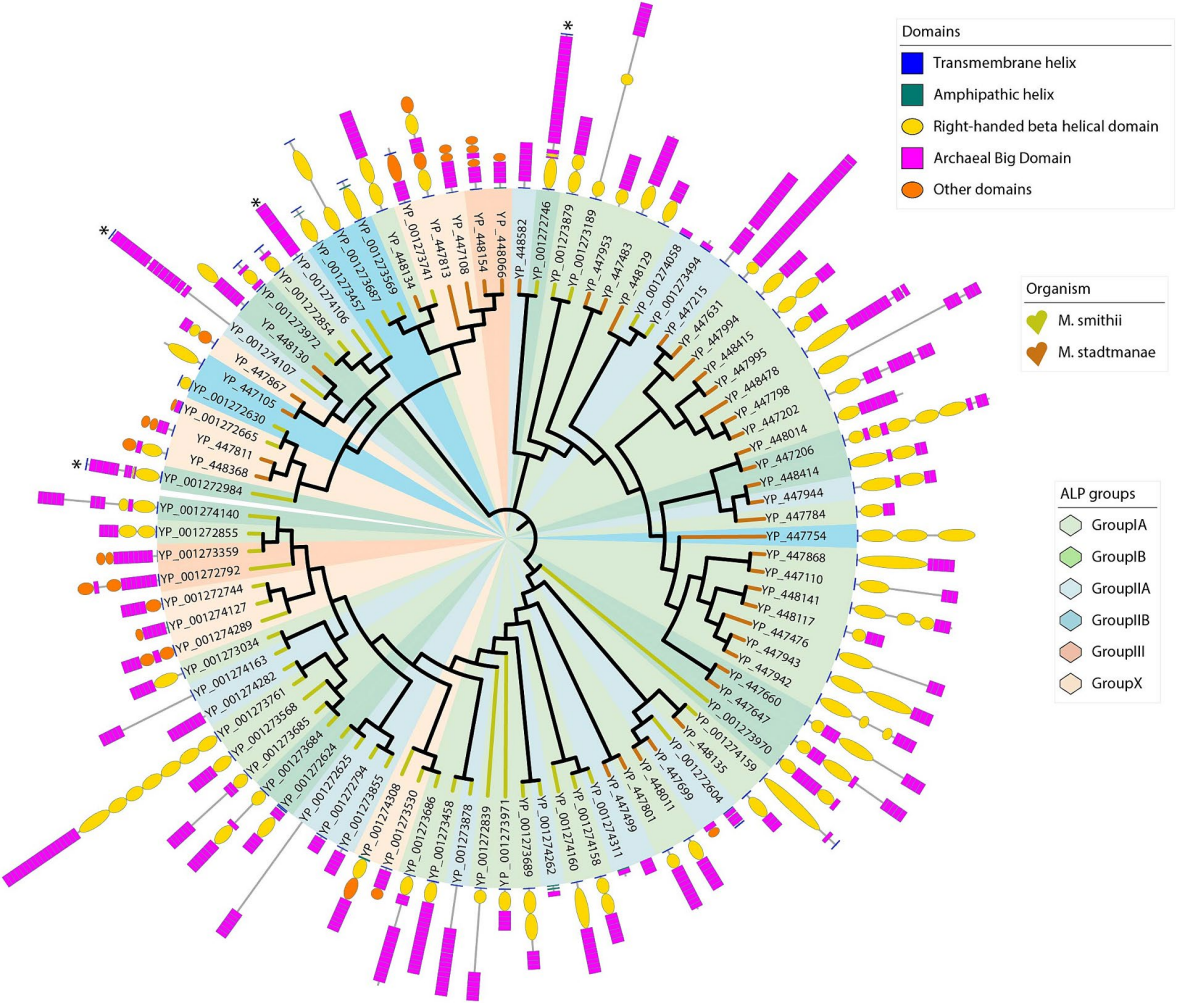
Phylogenetic tree of *Methanobrevibacter smithii* and *Methanosphaera stadtmanae* ALPs showing ALP groups based on domain architecture. These 1,000 bootstrapped neighbor-joining trees are constructed using 49 *M. smithii* ALPs and 42 *M. stadtmanae* full-length ALPs. The tree is displayed in circular mode with branch lengths ignored to keep the clarity of the display. Against each branch, the domain architecture of individual proteins is shown as described in the Methods section. Branches are labeled with the NCBI accession numbers of protein. The branches are colored for two methanogens, *M. smithii* (yellow) and *M. stadtmanae* (brown) similar to that of Figure 2. Five different domains have been marked as shown in “Domains” legend. Protein lengths are scaled in the tree display. Four ALPs are marked with an asterisk. These ALPs have ABD domains with nine β-strand Greek-key topologies. ALP groups and subgroups have been shown using six pastel colors as marked in “ALP groups” legend. The ALP groups do not form clusters in the phylogenetic tree because the tree is created from sequence-based clustering.

The alignment of ALPs with different repetitive structures of varying length may lead to misalignments, potentially introducing errors in phylogenetic inference. However, the detailed characterization of individual ALP domains allows to group different ALPs based on their specific domain architecture and independent of their sequence similarity (Figure 5). As an alternative approach, we have used density-based clustering of text strings that represent the domain architecture, which allows to bin ALPs into different distinct classes. Based on the clustering, we proposed five groups of ALPs in *M. smithii* and *M. stadtmanae*. A growing number of ALP groups might be expected in the future as more archaeal species are analyzed for divergent ALPs. The groups proposed here are based on the presence/absence and positions of ABD, RBH, transglutaminase, and other domains, as described further. In general, we observed that ALPs contain at least either the ABD or RBH domain. If the protein sequence did not have any of these domains, we did not classify it as ALP, although these proteins were picked up in our blast searches together with other ALPs. There were nine such sequences in *M. smithii* and five in *M. stadtmanae* (Supplementary Tables 5A,B). All ALPs of *M. stadtmanae* had a MAD only at the N-terminus (except three ALPs with no MAD), while 11 out of 49 *M. smithii* ALPs had MAD on both termini. Furthermore, five ALPs of *M. smithii* (NCBI accession: YP_001272625, YP_001272839, YP_001273878, YP_001274107, and YP_001274163) were not fully annotated for domains due to low sequence similarity with AlphaFold structures or partially predicted structures. Thus, they were tentatively assigned ALP groups based on current knowledge of their domains. The following three groups (excluding subgroups) and one currently uncategorized set of “others” ALPs are proposed in *M. smithii* and *M. stadtmanae* regardless of the position of the MAD, and they are listed in Supplementary Tables 6A,B.

#### Group-I

This is the largest ALP group in both *M. smithii* and *M. stadtmanae* (*n* = 52), and most proteins in this group consist of only three domain types, i.e., MAD, RBH domain, and ABD. No “Other” domain is present in this ALP group. The MAD is N-terminal in 48 ALPs, the only modifications being a duplication in one ALP (YP_001272624 of *M. smithii*) and an additional C-terminal MAD in two ALPs (YP_001272984 and YP_001273972 of *M. smithii*). Visual inspection of this group indicates that this group can be further divided into two subgroups:

*Subgroup-IA:* In most cases (*n* = 40), the RBH domain is adjacent to the MAD, followed by repeats of ABD domains in varying numbers. In general, the RBH domain is present as a single domain in the majority of ALPs (10 out of 16 *M. smithii* ALPs and 18 out of 24 *M. stadtmanae* ALPs). Several variations of this pattern are observed in this subgroup, e.g., multiple RBH domains can be present. A notable example is YP_001273761, which has seven RBH domain repeats at the N-terminus, followed by 18 ABD domain repeats. This is also the largest ALP found in *M. smithii* with a length of 4,691 amino acids. Compared to other subgroups, the ALPs of this group are larger.
*Subgroup-IB:* Similar to subgroup-IA, ALPs of this group also contain only ABD and RBH domains in addition to MAD; however, the relative positions of ABD and RBH domains are not fixed. In some ALPs, ABD repeats are found N-terminally to the RBH domain, e.g., YP_448130. Except for YP_001273972, all other ALPs of this group have a single MAD at the N-terminus. YP_001274127 has an amphipathic helix at the N-terminus, which could act as a membrane anchor as there is no TM domain. This ALP is also unique with a small RBH domain at the C-terminus and ABD domains at the N-terminus to RBH. *M. smithii* has seven and *M. stadtmanae* has five ALPs belonging to this group. No membrane anchor domain was located in YP_447953 of *M. stadtmanae*.

#### Group-II

Adhesin-like proteins of this group have only one domain, either ABD or RBH, present in them, in addition to a MAD at either or both terminals. Although such ALPs are present in our dataset (15 in *M. smithii* and five in *M. stadtmanae*), it might be possible that these are proteins with unidentified N-or C-terminal domains because of low sequence homology within the domains in AlphaFold structures. This is evident from the fact that four *M. smithii* ALPs of this group are partially annotated for domains. It is also possible that protein sequences of these ALPs are partial; for example, YP_001274262 has only one ABD in addition to two amphipathic helices possibly acting as MADs at the N-terminus, and it is only 203 amino acids long. Similarly, YP_001274311 of *M. smithii* is 156 amino acids long and has one amphipathic helix at the N-terminus and only one ABD domain.

*Subgroup-IIA*: ALPs of this subgroup (*n* = 13) are characterized by having only ABD, while RBH and ‘Other’ domains are completely absent. MAD is found N-terminal in eight ALPs, one ALP has C-terminal MADs, one on both termini, while in the other three ALPs, no MAD could be identified. ABD is present with a varying number of repeats, e.g., 1–17 repeats.
*Subgroup-IIB*: This group of ALPs has only the RBH domain and no other domain in addition to MAD. Five ALPs belong to this subgroup (four from *M. smithii* and one from *M. stadtmanae*). All ALPs from *M. smithii* of this group have a MAD on both termini, while *M. stadtmanae* ALP has one only N-terminally. Repeats of the RBH domain are present, which could mediate interactions by extending the length of ALPs.

#### Group-III [Transglutaminase (TG)-type]

Adhesin-like proteins in this subgroup have a transglutaminase domain at the C-terminus and ABDs at the N-terminus. Pfam also identified a pseudomurein-binding repeat domain beside the transglutaminase domain. Compared to other groups, this group has shorter ALPs. These ALPs have a single N-terminus transmembrane helix serving as a membrane anchor. Furthermore, there are no RBH domains in this group of ALPs, which probably points out that the RBH domain could also act as an adhesive domain in ALPs of other groups in addition to extending ALPs to reach the surface of other microorganisms. Currently, there are only four ALPs in this group from two organisms.

#### Others

There are seven *M. smithii* and six *M. stadtmanae* ALPs in our dataset that contain domains in addition to RBH, ABD, or transglutaminase domains (Table 2). These unique domains could be involved in specific substrate binding. YP_001273741 has MAD on both termini. The seven *M. smithii* domains have alpha–beta folds in the closest AlphaFold structures. Six *M. stadtmanae* ALPs are as annotated by Pfam. Furthermore, the four *M. smithii* ALPs (YP_001272746, YP_001272984, YP_001274106, and YP_001274107) with a longer ABD domain (with nine *β*-strands) are marked with an asterisk in a phylogenetic tree (Figure 5). YP_001272746 and YP_001272984 have ABD with a beta-strand extending from a loop of the RBH domain. These two ALPs have MAD on both sides. It is interesting to note that most ALPs in other groups, with MAD on both sides, have single domains and no repeats of ABD or RBH domains. Since these ABDs are structurally distinct from other ABDs, it might indicate the functional divergence acquired from other ALPs.

## Discussion

Nearly 20 years after their discovery, this study revisits the repertoire of adhesin-like proteins of *M. stadtmanae* and *M. smithii,* with a special emphasis on individual domain structures and overall domain architecture. The analysis is particularly aided by the recent advances in structure predictions, which has enabled us to identify and perform comparisons between different domain types and within domains, e.g., different ABD types. These findings allow us to define minimal ALPs more specifically, e.g., the presence of an ABD domain (and/or potentially also RBH), which are common features of the analyzed ALPs in these two organisms. Further analyses on other methanogens are required to determine if such a minimal criterion can identify ALPs in other organisms, especially those that are more distantly related, such as *Methanomassilicoccales*.

Using structure predictions has allowed us to characterize ALPs in much more detail but may also have some caveats. While AlphaFold offers insights into the potential folding of proteins, it is also known to have limitations. The inability to perform structure predictions of long proteins can be overcome, but it is currently more difficult to assess if the predicted protein may potentially also bind co-factors or ions. For example, for the Salmonella giant adhesin SiiE, a conserved sequence motif of five aspartates has been reported and experimentally verified to be involved in the binding of Ca^2+^. Calcium ions have been implicated in stabilizing and rigidifying the protein (Barlag and Hensel, 2015; Griessl et al., 2013; Guo et al., 2017). The same motif does not appear to be conserved in ABDs, but the presence of other conserved residues, such as aspartic acid in loops, indicates that the binding of ions, such as Ca^2+^, is reported here and may point toward similar mechanisms. Likewise, the potential presence of post-translational modifications in ABDs and other ALP domains may only be elucidated through experimental analyses of purified proteins.

Adhesin-like proteins are likely to play an important role under *in vivo* conditions, where growth substrates may be limited, and cell growth is more likely to occur in syntrophic and biofilm-like microbe–microbe interactions. The actual binding of ALPs to a substrate can currently also not be deduced from the structural predictions. Some of the bacterial big domains and the bacterial repetitive beta-helical domains have been experimentally verified to bind carbohydrates (Burnim et al., 2024). However, whether ALPs may function similarly remains to be experimentally investigated. The observed repetitive beta-helical folds point toward the binding of polymeric structures, such as polysaccharides (as has been proposed; Jenkins and Pickersgill, 2001), for bacterial proteins with these domains and have been identified for some proteins with similar folds, such as pectin lyase (Yoder et al., 1993). None of the currently known methanogens can produce methane from polysaccharides, indicating that the respective catalytic function of ALPs for direct polysaccharide degradation in methanogens is unlikely. However, RBH domains from methanogen ALPs may still fulfill important functions in methanogens as a mere binding/adhesive domain, which enables methanogens to adhere to other microorganisms that may provide substrates for methanogenesis. The proximity of methanogens to polysaccharide degraders would be beneficial as the fermentation of polysaccharides, such as pectin, would produce gaseous growth substrates (CO_2_ and hydrogen), which diffuse slowly through liquid matter (and in the presence of potentially competing microorganisms, such as sulfate reducers or acetogens). It would therefore be quite possible that ALPs, and specifically RBH domains, could mediate the adhesion of methanogens to such microorganisms, for example, through binding bacterial (or fungal/protozoan) cell-surface glycans. Glycan compositions may vary considerably between different species, and this may also partially explain the large repertoire of ALPs in the methanogen genome. ALPs could therefore be an interesting target to disrupt specific microbial interactions and reduce methanogen population that could account for reduced methane emissions or modulate the intestinal hydrogen metabolism in monogastrics.

Another explanation may be that ALPs may bind other polymeric/polysaccharide structures that do not directly contribute to increasing the substrate transfer between fermenters and methanogens. This could be the binding of ALPs to host-surface structure (host glycans) to mediate adhesion and retention of methanogens in the anoxic gut environment, or the binding to other symbiotic partners, such as Nanoarchaeota, which have recently been described for some methanogens, such as *Methanobrevibacter oralis* (Hassani et al., 2023). A recent study on extracellular vesicles (EV) may also be of interest in this regard (Weinberger et al., 2024). It was shown that EVs that are produced by gut methanogens are enriched in ALPs. ALPs may therefore potentially allow to direct EVs to specific interaction partners or link them to the cell the EV originated from, e.g., through ALPs with pseudomurein-binding domains.

Finally, it can also not be completely ruled out that some ALPs may bind to polysaccharides (or other polymers) found in organic matter. This may also help to reduce the distance between the methanogen and the polysaccharide degrader to enable the growth of the methanogens and/or to bind to the polysaccharide to last longer periods when growth conditions are not favorable (e.g., oxic) and metabolism of anaerobes is stopped or becomes dormant.

In conclusion, it can be stated that this bioinformatic analysis does provide novel insights into the structure and domain architecture of ALPs. However, it needs to be noted that many questions surrounding ALPs remain currently unanswered and may require more than computational analysis. This concerns not only the exact function and mechanism of these proteins but also the regulation of their expression as well as their evolution. The framework presented here for the proposed grouping of ALPs is a starting point for the classification of ALPs. The number of ALP groups is likely to increase as more genomes of methanogens are being analyzed in depth for ALP architectures, there are advancements in structural ALP analyses, and experimental verifications of the archaeal “adhesiome” functions are performed.

## Materials and methods

### Identification of complete sets of ALPs in *Methanobrevibacter smithii* and *Methanosphaera stadtmanae*

First, a raw dataset of putative ALPs was constructed by extracting protein FASTA sequences from an annotation-based search using keywords “adhesin-like protein” and “Asn/Thr-rich large protein” in 13 complete *Methanobacteriales* proteomes (Supplementary Table 7). This search picked 516 putative ALPs. These 516 ALPs were taken to query proteomes specifically of *M. smithii* and *M. stadtmanae* with the aim of identifying distantly related ALP sequences using the BLASTP algorithm with an e-value cutoff of 0.0001 (Altschul et al., 1990). All the sequence hits thus obtained were combined in a single FASTA file, separately for both species, and the resulting FASTA file contained 58 and 47 unique sequences belonging to *M. smithii* and *M. stadtmanae,* respectively.

### Domain annotations of ALPs

Each of the 105 ALPs of both organisms was queried against the AlphaFold database (Jumper et al., 2021; Varadi et al., 2021) via EBI’s “Sequence Similarity Search” tool^1^ to identify the closest structure based on sequence homology. In the first round of searches, we used full-length ALP sequences. Overall, 43 of 58 *M. smithii* ALPs and 32 of 47 *M. stadtmanae* ALPs could be matched to >90% identity for most of their sequence length, while 11 *M. smithii* and 13 *M. stadtmanae* ALPs matched to 40–90% identity. Structures based on the highest matching score were downloaded from the AlphaFold database. The amino acid sequences of the downloaded structures were individually aligned with the corresponding ALP sequence by Clustalx version 2.0 (Larkin et al., 2007) in order to locate the putative structural domains on ALP protein sequences. The remaining ALPs (five of 58 ALPs in *M. smithii* and eight of 47 ALPs in *M. stadtmanae*) had sequence identity between 20 and 30%, and thus identified domains for these ALPs were less reliable based on sequence homology to the nearest structures (Supplementary Tables 5A,B).

Since AlphaFold structures have currently a limitation of 2,700 amino acids for proteins, many ALP sequences could be annotated only partially for domains. For the remaining part of ALP sequences (partially annotated sequences that could not be annotated in the first round), we did another iteration of identifying the closest structures by querying only the unannotated part of the ALP sequence and keeping the identity cutoff to 40%. This allowed identifying further domains in ALPs and filled annotation gaps. In few cases, if at least one ABD was identified by AlphaFold, we were able to manually identify other ABD repeats. For example, the two closest AlphaFold structures (AlphaFold IDs: B9ACY6 and R7PVK4) were downloaded for an ALP (Accession: YP_001272746, length: 2879) from *M. smithii* that matched to >90% sequence identity. Only one ABD was present in B9ACY6, while six ABDs were located based on R7PVK4. However, we could locate another 17 ABD repeat domains similar to the ones located with the help of the nearest AlphaFold structures and extend the domains further at the terminus. Similarly, an AlphaFold structure (AlphaFold ID: B9AFH2) was matched to *M. smithii* ALP (Accession: YP_001273761) at 78% identity. The structure had only two RBH domains; however, we could locate five more RBH domain repeats on this ALP sequence. We have also marked these manually identified domains in Figure 5 together with other domains.

In rare cases where domains were not identified by AlphaFold, we took annotations from Pfam to improve the domain identifications. InterProScan searches (Blum et al., 2021; Paysan-Lafosse et al., 2022) were carried out for all ALPs. The sequences of domains identified by Pfam were aligned with annotated domains in other ALPs, followed by manually extending the domain boundaries on both sides. Most Pfam predictions agreed with AlphaFold predictions except ABD, which was assigned bacterial Ig-like domains (Pfam IDs: PF16640, PF02369, and PF02368) by Pfam. This could be because the ABD domain definition is missing in the UniProt database. Pfam was also useful for assigning functional annotation for AlphaFold domains in ALPs, as listed in Table 2.

The transmembrane helices were identified by TMHMM^2^ (Hallgren et al., 2022) and confirmed by the presence of helical structures in AlphaFold structures. HeliQuest was used to predict amphipathic helix with analysis window size taken as 18 amino acids (Gautier et al., 2008). In general, hydrophobicity values of approximately 0.5 indicate the possibility of forming an amphipathic helix. The PDB files were displayed using ChimeraX (Pettersen et al., 2021).

### Sequence alignments, phylogenetic analysis, and sequence logos

All multiple sequence alignments were created by Clustal version 2.0 (Larkin et al., 2007; Thompson et al., 1997) using default parameters. The phylogenetic tree was calculated using the neighbor-joining method (Saitou and Nei, 1987) as implemented in Clustal v2.0, based on the multiple sequence alignment and 1,000 bootstrap trials to confirm the robustness of branches (Felsenstein, 1985). The trees were displayed by iToL v6.8^3^ (Letunic and Bork, 2021). The sequence logo was created by WebLogo^4^ using a user-defined coloring scheme.

We also downloaded 82 bacterial stalk domain sequences from the Pfam database as per the accessions given in (Monzon et al., 2021) representing 3,542 bacterial fibrillar adhesins. These belong to the ESET clan and others. The pairwise sequence comparisons of 80 stalk domains of bacteria and major clades of ABD domains were carried out using Clustal Omega v1.2.4 (Sievers et al., 2011) in clustering mode. The pairwise distance matrix was calculated and converted into percent identities. The percent identity values were averaged across ABD clades and other groups and mentioned in Table 3 separately for two organisms.

**Table 3:** Average % identities within and across ABD clades with bacterial stalk domains.

	Clade-I ABD	Clade-II ABD	Clade-III ABD
Clade-I	26.4 (*M. smithii*-32, *M. stadtmanae*-34)		
Clade-II	*M. smithii*-16.8, *M. stadtmanae*-17	19.7 (*M. smithii*-27, *M. stadtmanae*-20)	
Clade-III	*M. smithii*-15.2	*M. smithii*-16.6	*M. smithii*-31.9
Bacterial stalk	14.2	14.5	14.8

### Density-based clustering of ALPs based on domain architecture

Adhesin-like protein domains were abbreviated by single letter codes (TMD = t, RBH = r, ABD = a, pseudomurein-binding domain = b, transglutaminase = g, OTHER = o), and domain sequences of individual ALPs were transcribed into strings of letters (e.g., sequence TMD-RBH-ABD-ABD-ABD-ABD-ABD = traaaaa). The DBSCAN library in R was used to perform density-based clustering of the string. The analysis was run using default parameters. Clusters were defined by an epsilon (eps) of 0.5 and minimum number of points (minPts) as 1.

## Data Availability

The original contributions presented in the study are included in the article/Supplementary material, further inquiries can be directed to the corresponding author.

## Footnotes

^1^https://www.ebi.ac.uk/Tools/sss/fasta/

^2^https://services.healthtech.dtu.dk/services/TMHMM-2.0/

^3^https://itol.embl.de/

^4^https://weblogo.berkeley.edu/logo.cgi
