# Supplementary material for: Structural and functional insights from the sequences and complex domain architecture of adhesin-like proteins from Methanobrevibacter smithii and Methanosphaera stadtmanae
Source: Front Microbiol. 2024 Oct 21;15:1463715. doi: 10.3389/fmicb.2024.1463715 (PMC11532034; doi:10.3389/fmicb.2024.1463715)

Supplementary Table1- List of AlphaFold accessions used for domain annotations for *M. smithii* and *M. stadtmanae*.

M. smithii				***M. stadtmanae***		
**S.N.**	**AlphaFold DB:ID**	**% Identity**		**S.N.**	**AlphaFold DB:ID**	**% Identity**
1	A5UJ58	100		1	Q2NI46	100
2	A5UJ78	100		2	Q2NID0	100
3	A0A2H4U5L0	35		3	A0A2A2HE23	45
3	A0A219ANP1	31		3	A0A328Q5W0	49
4	A5UJ84	100		4	A0A1D2WFU6	100
5	A0A843LUC5	100		5	A0A328PYK6	97
6	A5UJF5	100		5	A0A328Q5W0	99
7	A0A1D2WSE0	100		5	A0A328Q6U3	31
8	A5UJJ8	100		6	A0A832RK30	30
9	3 REPEATS	98		6	A0A1D2X3L9	28
9	R7PVK4	100		7	A0A328Q0F6	99
10	A0A1G5XC85	45		7	A0A328QAI7	99
10	A0A843MAU4	99		8	Q2NHL9	100
11	A5UJP8	100		9	A0A2A2HE23	52
12	A5UJS6	100		9	A0A328Q7L2	30
13	A0A843M7R0	92		10	A0A328Q9M3	98
14	A0A1D2WTP5	100		10	Q2NFU3	48
15	A5UJV9	100		11	Q2NH47	100
16	A5UK14	100		12	A0A328Q569	99
17	A5UK78	100		12	A0A328Q0F6	48
18	A0A843I939	48		13	A0A2A2HER1	51
19	A0A1D2WV90	100		13	A0A2A2HEM6	41
20	A5UKD8	100		13	A0A2A2HDJ6	30
21	A0A843MAF3	100		14	A0A2A2HER1	45
22	A0A843LTW4	77		14	A0A2A2HEM6	35
22	D2ZR10	39		14	A0A2A2HDJ6	29
23	A0A1D2WSY5	100		15	Q2NGJ7	100

24	A5ULL1	100		16	A0A2A2HE23	41
25	A0A1D2WRA4	57		16	A0A1D2X1A8	27
25	A0A328S9P1	39		17	A0A1D2WJK0	100
26	A5ULP8	100		18	Q2NGA9	100
27	A0A1D2WV05	100		19	A0A2A2HE23	51
28	A0A1D2WV51	100		19	A0A328Q5W0	97
29	A0A1D2WWA8	100		20	Q2NG95	100
30	A0A843I8I4	41		21	Q2NG85	100
30	R7PV57	97		22	Q2NG84	100
31	D2ZPN6	47		23	A0A1D2W7A3	100
31	A0A843F5F9	40		23	A0A2A2HFS5	37
32	A0A843F0H3	51		24	Q2NG29	100
32	A0A6A8RQ03	25		25	A0A2A2HE23	44
33	A0A843F6M5	47		25	A0A843HFD5	33
33	A0A843EZ93	52		26	A0A1D2W849	100
34	R9SMV4	35		27	A0A2A2HE23	54
35	A5UME5	100		27	A0A1D2X356	25
36	3 REPEATS	78		28	A0A2A2HE23	53
36	R7PX92	97		28	A0A1D2X3L9	27
37	A5UMQ9	100		29	A0A328SGM0	44
38	A0A1D2WRA4	100		29	A0A328RVP0	27
38	A0A1D2X1A8	28		30	Q2NFU3	100
39	A0A315XKD0	34		31	Q2NFQ2	100
39	B9AFG9	49		32	A0A1D2WIH3	100
40	A0A843DA90	50		33	Q2NFN5	100
41	A0A1D2WQM3	100		34	A0A166EX48	29
42	A5UN26	100		35	Q2NFI0	100
43	B9ADZ7	100		36	Q2NFC9	100
44	A5UNG0	100		37	A0A1D2WC44	100
45	R7PX74	91		38	Q2NFB6	100
45	A0A3A5HKF6	32		39	A0A843HHL6	56
46	A5UNI1	100		39	A0A843H9Z3	35

47	A0A843M403	99		40	A0A1D2WIF1	100
47	A0A843M1H4	40		41	A0A328Q7U5	99
48	A5UNL2	100		41	A0A328Q7N9	99
49	A0A1D2WTQ2	99		42	Q2NF92	100
49	A0A1D2X3S3	34		43	A0A1D2W969	100
49	A0A843AMJ8	28		44	A0A328SGM0	45
50	A0A219ANQ0	29		44	A0A328RV88	28
50	A0A166F7I3	25		45	A0A328Q7L2	95
50	A0A1D2WTP3	30		45	A0A328PYW6	68
51	D2ZPN6	46		46	A0A328Q6U3	71
51	A0A1D2X1A8	27		46	A0A328Q5W0	97
52	A0A1D2WR37	100		46	A0A328PYW6	94
53	A5UNW6	100		47	Q2NE14	100
54	A5UNY6	100				
55	A5UNZ3	100				
56	A0A843LJQ7	99				
56	A0A843LJ92	94				
57	A5UP15	100				
58	A0A1D2WX41	100				

Supplementary Table2: Short hydrophobic helices that can potentially insert into the cell membrane.

**S.N.**	**Accession**	**Amino-acid Sequence**	**Length**	**Hydrophobicity index**
1	YP_001272630	LITALIVALLSLSTVGAV	18	0.952
2	YP_001272792	MIFALMILTVFLSVSAVSAM	20	1.064
3	YP_001272794	IVLILLLTLALFSISNVSA	19	1.093
4	YP_001273359	MLGSVCAA	8	*
5	YP_001273457	MLFFVIMGCVSA	12	*
6	YP_001273569	LKILFLGLLLVLCVNSVSAA	20	1.057
7	YP_001273684	MMLVSMLLLAILTLGAVSA	19	1.052
8	YP_001273686	MLFLIILSSISLASAS	16	*
9	YP_001273687	MLILVMICCFILSLSAVSAI	20	1.231
10	YP_001273879	MLICSIGAVNAT	12	*
11	YP_447108	MSMILLGISAVSA	13	*
12	YP_448130	MLISISSVTAS	11	*
13	YP_448154	MILMFLMTLIISITAVSAA	19	1.114
14	YP_448368	IFPIFIILTLLLCISTVSAS	20	1.294

*Values could not be calculated as HeliQuest requires at least 18 amino acids long peptide.

Supplementary Table3- N-terminal sequences of *M. smithii* and *M. stadtmanae* ALPs are abundant in charged residues such as Asparagine (N), Lysine (K) and, Arginine (R) together with other amino-acids with long hydrophobic side chains (coloured in black).

YP_447483	MNIMSTNK
YP_447631	MHKNR
YP_447647	MKNKN
YP_447660	MIKMKNK
YP_447754	MKNK
YP_447784	MTKKN
YP_447798	MFKKYSK
YP_447105	MEENLIKIKK
YP_447801	MKINNK
YP_447813	MNKK
YP_447867	MKNK
YP_447868	MDKNVKK
YP_447942	MKNK
YP_447943	MKNK
YP_447944	MKNK
YP_447994	MLNKK
YP_447995	MNKN
YP_448011	MNMKKN
YP_448014	MKINNKN
YP_448117	MFKKYSK
YP_448129	MKK
YP_448134	MKTINK
YP_447110	MNNNIK
YP_448135	MYNKR
YP_448141	MFKKYSK
YP_448368	MKR
YP_448414	MIKMKNK
YP_448415	MRNQK
YP_448478	MRNQK
YP_448582	MKIIK
YP_447202	MFKKYNKE
YP_447206	MITRNK
YP_447215	MVNK
YP_447476	MKNK
YP_001272604	MNKIKY
YP_001272794	MKLKK
YP_001272839	MMIKKR
YP_001272854	MIEKKYKK
YP_001272855	MNKK
YP_001272984	MFKNKQK
YP_001273034	MFKDKF
YP_001273189	MDKK
YP_001273458	MKHGRL
YP_001273494	MDNK
YP_001273530	MKNLIFKK
YP_001273568	MNFKY

YP_001273569	MNFK
YP_001273684	MKLNK
YP_001273685	MRLNK
YP_001273689	MVKNIK
YP_001273741	MIRKIN
YP_001273761	MSLKKN
YP_001273855	MIFFCDIMKFNK
YP_001273878	MKNRK
YP_001273970	MIFIINHIAIKYEVNNMNKK
YP_001273971	MKINNK
YP_001273972	MNNLK
YP_001274058	MNKEN
YP_001274106	MNYLK
YP_001274140	MKFNKQ
YP_001274158	MRCFMRKK
YP_001274159	MRFNKC
YP_001272665	MKKDKKK
YP_001274160	MSNFK
YP_001274163	MLRRNKNLKNK
YP_001274282	MEYNNSENGKN
YP_001274289	MNVKNK
YP_001272744	MFNKK
YP_001272746	MRKFFEFYNWCVDKMVKK

Supplementary Table4- C-terminal sequences of *M. smithii* and *M. stadtmanae* ALPs are abundant in charged residues such as Asparagine (N), Lysine (K) and, Arginine (R).

YP_001273569	NKR
YP_001273741	QSVANKYR
YP_001272604	KRK
YP_001272854	KRKNKM
YP_001272746	RKRKL
YP_001274107	SRRKK
YP_001272630	KRE
YP_001274159	GYKRNSKEEDE
YP_001273457	KRRKLNEE
YP_001273687	KRRKDRENE
YP_001272984	KKR
YP_001273972	NDIKSMIEKKNGK

Supplementary Table5(a)- List of accessions of proteins in *M. smithii* that were not classified as ALPs as they lack ABD or RHB domain.

**Accession**	**Annotation**	**Length**	**Domain identified****	**TM**	**Amphipathic helix**
YP_001272732	adhesin-like protein	153	Lectin like	0	1
YP_001272822	Hypothetical protein	239	Zinc-ribbon domain	1	0
YP_001272910	putative adhesin-like protein	71	Seems partial protein fragment	0	0
YP_001272974	surface protease	247	alpha/beta fold	0	1
YP_001272985	putative adhesin-like protein	376	alpha/beta fold	2	0
YP_001273153	putative adhesin-like protein	262	alpha/beta fold	1	0
YP_001274260	hypothetical protein	847	alpha/beta fold	0	2
YP_001274341	hypothetical protein	150	0	1	0
YP_001272701	Hypothetical protein	238	alpha/beta fold	1	0

Supplementary Table5(b)- List of accessions of proteins in *M. stadmanae* that were not classified as ALPs as they lack ABD or RHB domain.

**Accession**	**Annotation**	**Length**	**Domain identified****	**TM**	**Amphipathic helix**
YP_447181	hypothetical protein	300	alpha/beta fold	1	0
YP_447257	hypothetical protein	412	beta propeller	2	0
YP_447787	hypothetical protein	263	alpha/beta fold	1	0
YP_447812	asn/thr-rich large protein family	421	alpha/beta fold	1	0
YP_447885	hypothetical protein	225	alpha/beta fold	1	0

** The domains were identified based on the nearest homologous structure in AlphaFold database.

Supplementary Table6(a)- Classification of ALPs of *M. smithii*

S.N.	Accession	Length	TM	Amphipathic helices	ALP group
1	YP_001273686	2101	0	-	IA
2	YP_001273879	1430	0	-	IA
3	YP_001272839	2065	1	-	IA
4	YP_001272855	1049	1	-	IA
5	YP_001273034	1026	1	-	IA
6	YP_001273189	3684	1	-	IA
7	YP_001273458	1730	1	-	IA
8	YP_001273568	951	1	-	IA
9	YP_001273685	1909	1	-	IA
10	YP_001273689	1491	1	-	IA
11	YP_001273761	4691	1	-	IA
12	YP_001273971	767	1	-	IA
13	YP_001274158	1262	1	-	IA
14	YP_001274160	1702	1	-	IA
15	YP_001272854	592	2	-	IA
16	YP_001274159	1831	2	-	IA
17	YP_001273684	1941	0	-	IB
18	YP_001273970	1370	1	-	IB
19	YP_001274140	2193	1	-	IB
20	YP_001272624	1022	2	-	IB
21	YP_001273972	649	2	-	IB
22	YP_001272625	2530	0	-	IIA
23	YP_001272794	336	0	-	IIA
24	YP_001274262	203	0	2	IIA
25	YP_001274282	748	0	-	IIA
26	YP_001274311	156	0	1	IIA
27	YP_001272630	251	1	-	IIB
28	YP_001273457	755	1	-	IIB
29	YP_001273494	202	1	-	IIA

30	YP_001273569	796	1	1	IIB
31	YP_001273687	1879	1	-	IIB
32	YP_001273855	532	1	-	IIA
33	YP_001273878	2036	1	-	IIA
34	YP_001274058	128	1	-	IIA
35	YP_001274106	1152	1	-	IIA
36	YP_001274107	2710	1	-	IIA
37	YP_001274163	1884	1	-	IIA
38	YP_001272604	354	2	-	IIA
39	YP_001272746	2879	2	-	IIB
40	YP_001272984	1414	2	-	IB
41	YP_001272792	1496	0	-	III
42	YP_001273359	1112	0	-	III
43	YP_001274127	612	0	1	X
44	YP_001274308	1434	0	1	X
45	YP_001272665	203	1	-	X
46	YP_001272744	770	1	-	X
47	YP_001273530	620	1	-	X
48	YP_001274289	1007	1	-	X
49	YP_001273741	1055	2	-	X

Supplementary Table6(b)- Classification of ALPs of *M. stadtmanae*

S.N.	Accession	Length	TM	Amphipathic helices	ALP group
1	YP_447110	1909	1	-	IA
2	YP_447202	2120	1	-	IA
3	YP_447476	2044	1	-	IA
4	YP_447483	1552	1	-	IA
5	YP_447631	2979	1	-	IA
6	YP_447784	706	1	-	IA
7	YP_447798	3356	1	-	IA
8	YP_447801	1222	1	-	IA
9	YP_447868	1790	1	-	IA
10	YP_447942	1965	1	-	IA
11	YP_447943	2037	1	-	IA
12	YP_447953	973	0	-	IA
13	YP_447994	1104	1	-	IA
14	YP_447995	769	1	-	IA
15	YP_448011	1063	1	-	IA
16	YP_448014	1311	1	-	IA
17	YP_448117	735	1	-	IA
18	YP_448129	857	1	-	IA
19	YP_448134	1921	1	-	IA
20	YP_448135	853	1	-	IA
21	YP_448141	1896	1	-	IA
22	YP_448415	1324	1	-	IA
23	YP_448478	2214	1	-	IA
24	YP_447206	2774	1	-	IB
25	YP_447647	2459	1	-	IB
26	YP_447660	2468	1	-	IA
27	YP_447944	1519	1	-	IB
28	YP_448130	1140	0	-	IB
29	YP_447105	816	1	-	IIB

30	YP_447215	2036	1	-	IIA
31	YP_447499	265	0	-	IIA
32	YP_447754	2151	1	-	IIB
33	YP_448582	843	1	-	IIA
34	YP_448414	1447	1	-	IB
35	YP_448066	640	0	1	III
36	YP_448154	976	0	-	III
37	YP_447108	881	0	-	X
38	YP_447699	251	0	-	X
39	YP_447811	616	0	-	X
40	YP_447813	1875	1	-	X
41	YP_447867	727	1	-	X
42	YP_448368	834	0	-	X

Supplementary Table7- List of 13 complete *Methanobacteriales* proteomes used to obtain initial dataset for ALPs

Methanobrevibacter_ruminantium (NC_013790)

Methanobrevibacter millerae (CP011266)

Methanobrevibacter olleyae (CP014265)

Methanobrevibacter smithii ATCC_35061 (NC_009515)

Methanosphaera stadtmanae DSM_3091 (NC_007681)

Methanobrevibacter sp. AbM4 (NC_021355)

Methanobacterium formicicum (CP006933)

Methanobacterium paludis (NC_015574)

[Methanothermobacter thermautotrophicus str. Delta H (NC_000916)

Methanothermobacter marburgensis (NC_014408)

Methanothermus fervidus (NC_014658)

Methanobacterium sp. MB1 (NC_023044)

Methanobacterium lacus (NC_015216)

Supplementary Figure1: Helical wheel diagrams of amphipathic helices (Non-TM membrane anchor domains) identified in *M. smithii* and *M. stadtmanae* ALPs

The figures were displayed with HeliQuest. The hydrophobicity values above 0.5 indicate higher presence of hydrophobic residues. In these amphipathic helices, hydrophobic residues were on one side of wheel while other side shows presence of hydrophilic residues. Threonine and Serine are small polar amino acids which frequently occur in TM helices also.

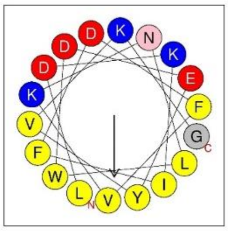

YP_001273569 (*M. smithii*)
LDKIFDFVKNLWDEYVKGKNSVK
Hydrophobicity: 0.439

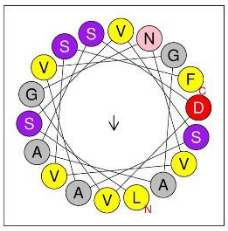

YP_001274127 (*M. smithii*)
LSVSAVGAASDVGNVVSF
Hydrophobicity: 0.499

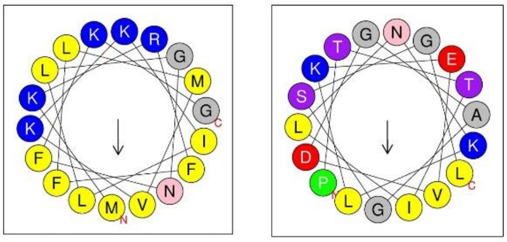

YP_001274262 (*M. smithii*)
MKRFFLMVKKILLGNFKG (Hydrophobicity: 0.577)
IETPTTILNKLKEVDGAGSGLNA (Hydrophobicity: 0.313)

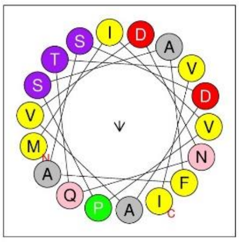

YP_001274308 (*M. smithii*)
MIVPSAFASDAVDNQTVISSDEQIEISAV
Hydrophobicity: 0.542

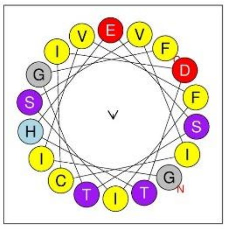

YP_001274311 (*M. smithii*)
MGIVFISVICIDTHESTGFSV
Hydrophobicity: 0.773

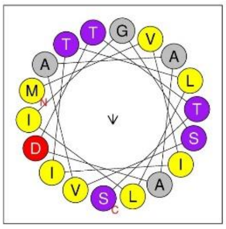

YP_448066 (*M. stadtmanae*)
MVIITLLIGSVAAADTTS
Hydrophobicity: 0.741

Supplementary Figure2: Distribution of ABD domain length across *M. smithii* and *M. stadtmanae* ALPs

ABDs had variable length and majority of them in both species are between 80-100 amino acid long. ABDs in *M. stadtmanae* are slightly longer as compared to *M. smithii*.

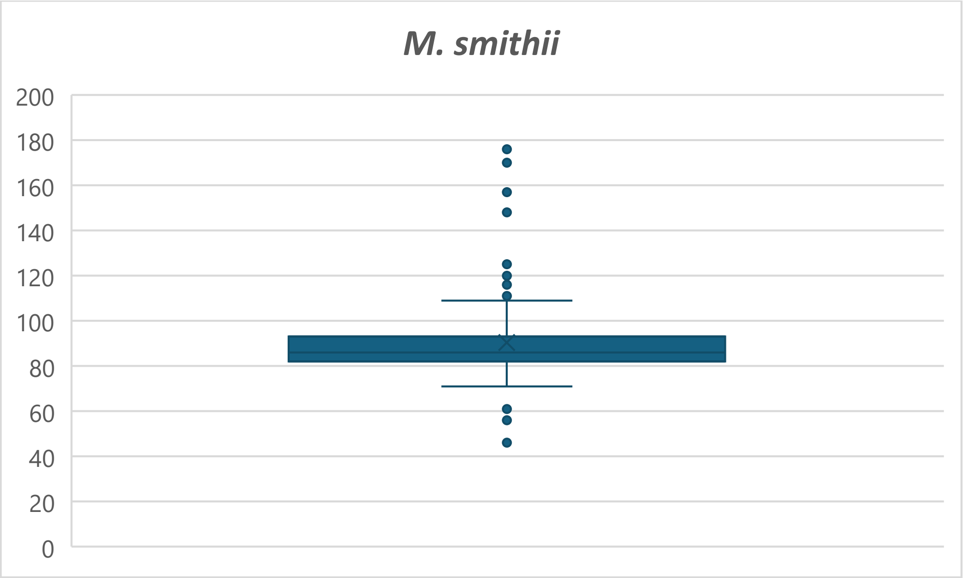

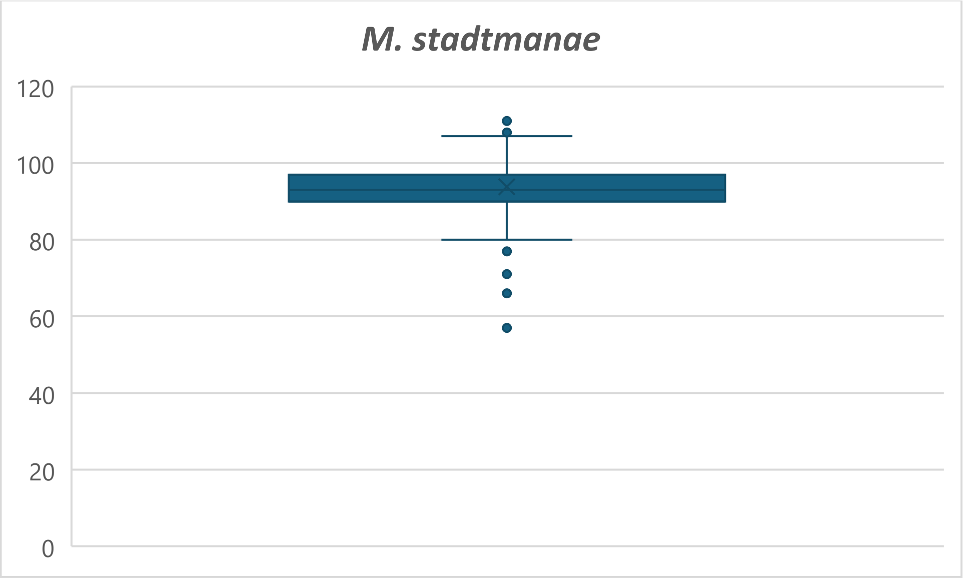

Supplement: Supplementary file 1 [file Data_Sheet_1.PDF]
